# Effectiveness of Follow-Up Mass Vaccination Campaigns Against Measles and Rubella to Mitigate Epidemics in West Africa (2024–2025): A Cross-Sectional Analysis of Surveillance and Coverage Data

**DOI:** 10.3390/vaccines14010075

**Published:** 2026-01-09

**Authors:** Marcellin Mengouo Nimpa, Ado Mpia Bwaka, Felix Amate Elime, Milse William Nzingou Mouhembe, Adama Nanko Bagayoko, Edouard Mbaya Munianji, Christian Tague, Joel Lamika Kalabudi, Criss Koba Mjumbe

**Affiliations:** 1 World Health Organization, Regional Office for Africa, Brazzaville 242, Congo; bwakaa@who.int (A.M.B.); amate76@yahoo.com (F.A.E.); milse.nzingoumou@who.int (M.W.N.M.); 2 McKing Consulting Corporation, 2900 Chamblee Tucker Rd, Atlanta, GA 30341, USA; bagayokoad@who.int; 3 Faculty of Public Health, University of Kananga, Kananga 243, Democratic Republic of the Congo; edombaya7@gmail.com; 4 Faculty of Medicine, Université Libre des Pays des Grands Lacs, Kyeshero Lusaka Rue, 218, Goma 243, Democratic Republic of the Congo; 5 Department of Communication, Faculty of Arts and Humanities, University of Kinshasa, Kinshasa 243, Democratic Republic of the Congo; joellamika@gmail.com; 6 Department of Public Health, Faculty of Medicine, University of Lubumbashi, Lubumbashi 243, Democratic Republic of the Congo; koba.mjumbe@unilu.ac.cd

**Keywords:** measles, rubella, vaccination campaigns, epidemics, West Africa, coverage surveys

## Abstract

Background/Objectives: Despite large-scale measles and rubella (MR) vaccination campaigns in West Africa, measles outbreaks persist, raising concerns about campaign effectiveness, coverage, and underlying determinants. This study assesses the impact of MR follow-up campaigns in 12 of 17 West African countries (2024–2025) and examines the factors contributing to post-campaign outbreaks. The main objective of this study is to evaluate the impact of MR campaigns on measles transmission, identify the characteristics of post-campaign outbreaks, and propose strategies to improve campaign effectiveness and accelerate progress toward measles elimination in West Africa. Methods: We conducted a cross-sectional and ecological analytical study to examine spatial and temporal variations based on measles surveillance data from 2024 to 2025, post-campaign coverage surveys (PCCS), district-level outbreak reports, and administrative coverage reports. Trends in measles cases before and after the MMR campaigns were assessed, along with demographic characteristics and spatial analyses of confirmed cases. Results: In 2024, 70.5% (12/17) of countries conducted measles vaccination campaigns, but measles outbreaks increased in 2025 (64 districts in 2024 versus 383 in 2025). Children under five remained the most affected (54%), with 85% of cases being either unvaccinated (57%) or of unknown status (28%). Administrative coverage exceeded 95% in most countries, but measles PCCS revealed gaps, with only Senegal (93%) and Guinea-Bissau (94%) achieving high verified coverage. No country achieved 95% national MPCC. Conclusions: Suboptimal campaign quality, gaps in immunity beyond target age groups, and unreliable administrative data contributed to the persistence of outbreaks. Recommendations include extending Measles vaccination campaigns to older children (5–14 years), improving preparedness by drawing on experiences from other programs such as polio, standardizing PCCS data survey and analysis methodologies across all countries, and integrating Measles vaccination campaigns with other services such as nutrition.

## 1. Introduction

### 1.1. Global and Regional Burden of Measles

Measles remains one of the most contagious and deadly vaccine-preventable human diseases in the world, with a basic reproduction number (R0) of 12 to 18, making it significantly more transmissible than SARS-CoV-2 (R0 2–3) or Ebola virus (R0 1.5–2.5) [1], with around 20 million cases per year and significant mortality among children under five years of age [2].

Despite the availability of a safe and effective measles and rubella (MR) vaccine since 1963, measles caused approximately 140,000 deaths worldwide in 2022, primarily among children under five in low-income countries, and outbreaks continue to occur, particularly in areas with suboptimal vaccination coverage or where supplementary immunization activities (SIAs) are delayed [2]. The African Region accounted for 38% of global cases between 2021 and 2023, with West Africa experiencing cyclical outbreaks every 2 to 5 years [3]. The WHO African Region has made substantial progress toward measles elimination, but persistent immunity gaps and uneven campaign quality have hampered sustained success.

The Global Vaccine Action Plan (GVAP) aimed for the elimination of measles in five WHO regions by 2020, but only the Americas maintained their elimination status. Africa’s elimination deadline was extended to 2030, but the region reported a 400% increase in cases between 2022 and 2024, with Nigeria alone accounting for 25% of continental cases [4]. This resurgence has been attributed to three main factors:

1. Vaccination gaps: 22 million children worldwide did not receive their first dose of measles vaccine in 2022, with West Africa contributing 3.2 million of these [5];
2. Disruptions related to COVID-19: 61% of African countries reported postponements of measles vaccination campaigns during the 2020–2021 period, creating an immunity debt [6];
3. Urban–rural disparities: Coverage in urban slums in West Africa (e.g., Lagos, Abidjan) is 15–20% lower than national averages due to mobility and documentation problems [7]. Eliminating Measles Rubella remain a key goal of Immunization Agenda 2030 (IA2030).

In 2024, 12 of the 17 countries in the WHO West Africa inter-Country Support Team (IST-WA) conducted measles follow-up campaigns targeting different age groups. However, by mid-2025, measles outbreaks had been observed, raising concerns about the effectiveness of these campaigns. Recent studies have questioned the reliability of administrative vaccination coverage data and highlighted the limitations of using national vaccination coverage as the sole predictor of outbreak risk [8]. Furthermore, the data suggest that even countries with coverage > 95% can experience outbreaks due to factors such as delayed campaign timing, poor cold chain management, and waning immunity [9]. Countries with national coverage above 95% may still experience outbreaks due to spatial heterogeneity and pockets of low coverage. This phenomenon has been well documented, including in a landmark study by Gustafson et al. (NEJM, 1987), showing that measles outbreaks can still occur despite vaccine coverage ≥ 95% in school-aged populations [10].

### 1.2. Vaccination Strategies and Gaps in Evidence

The WHO recommends 95% vaccination coverage with two doses through routine immunization and community-based vaccination (CBV) to achieve herd immunity. However, systematic reviews indicate that the effectiveness of measles vaccination campaigns in Africa is variable.

- Temporal immunity: Case–control studies in Niger (2023) showed a vaccine efficacy (VE) of 92% at 6 months after the campaign, decreasing to 78% at 24 months due to decreased immunity in malnourished children [11];
- The limitations of age-based targeting: A meta-analysis of 12 supplementary immunization activities (2015–2024) revealed that campaigns limited to children under 5 left 28 to 42% of school-aged children vulnerable, thus fueling epidemics among 5- to 14-year-olds [12];
- Data reliability: Administrative coverage overestimates actual protection by 8 to 25% compared to PCCS, as has been documented in Ghana (admin: 97% vs. PCCS: 82%) and Burkina Faso (admin: 95% vs. PCCS: 74%) [13,14].

### 1.3. Justification of the Study and Innovation

The objective of this study was to:

- Triangulate multiple data streams (surveillance, PCCS, epidemic lineages) to assess the campaign’s impact beyond routine coverage measures [15];
- Assess age-specific immunity gaps through stratified case analysis in 5- to 14-year-olds, a demographic group often excluded from campaign targets;
- Propose context-specific solutions, informed by the unique challenges of West Africa (e.g., nomadic populations, conflict zones).

## 2. Materials and Methods

### 2.1. Study Design and Framework

We conducted a cross-sectional and ecological analytical study to examine the spatial and temporal variations in measles epidemics in 17 West African countries from January 2024 to April 2025, focusing on the 12 countries that implemented MMR follow-up campaigns in 2024.

### 2.2. Data Sources

We collected data from the following sources:

- Measles surveillance data based on cases (2024–2025);
- Laboratory-confirmed cases (IgM+);
- Surveillance data: Lists of lines with demographic and vaccination status and epidemiologically linked cases, weekly case reports from the Inter-Country Support Team West Africa surveillance system, etc.;
- Vaccination coverage data: Administrative coverage reports of national immunization programs and post-campaign coverage surveys (PCCS);
- Surveillance data: Weekly case reports from West African countries surveillance system to WHO inter-Country Support Team;
- Epidemic measures: District-level attack rate, age-stratified incidence, spatial clustering analysis, etc.

All this data is freely accessible on the corresponding websites, which guarantees the confidentiality and ethics of the data, given that it is aggregated.

The measles–rubella vaccines used across West African countries during the 2024 follow-up campaigns were WHO-prequalified lyophilized MR vaccines, primarily Serum Institute of India (MR-VAC) and GSK/Takeda formulations, depending on country procurement. All MR vaccines require storage between +2 °C and +8 °C and must be protected from light. Reconstituted vials must be discarded after 6 h according to WHO guidelines.

### 2.3. Analysis

We analyzed trends in confirmed measles cases per week, demographic distribution (age, sex, vaccination status), spatial distribution of outbreaks, comparison of administrative coverage and PCCS coverage, correlation between districts with low coverage and outbreak clusters, and comparisons of incidence before and after the campaign.

### 2.4. Exploratory Analysis

#### 2.4.1. Mapping

**Figure 1:**
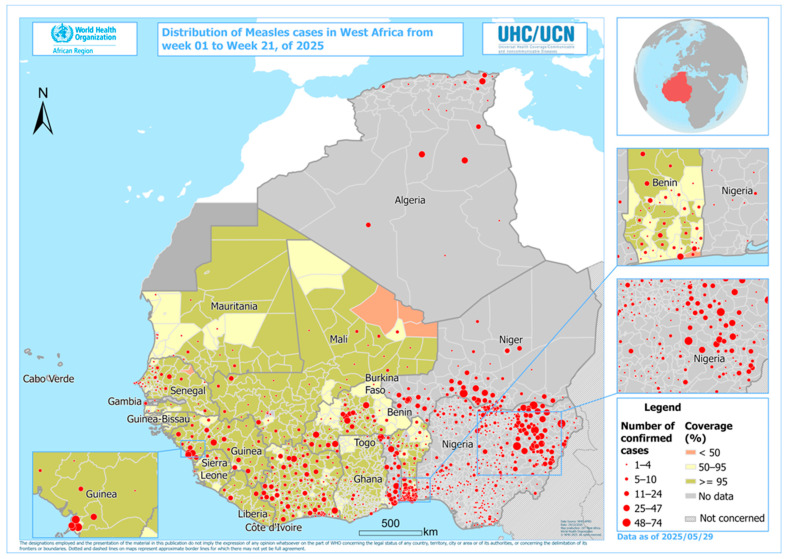
Clusters of Measles confirmed cases in West African countries, week 1–22 of year 2025.

The data mapping was performed using QGIS version 3.22.16. To do this, we imported the two layers from the WHO Afro website using the WHO 2024 projection and the .shp extension. One of the layers imported into our software represented a map subdivided by country, while the other represented the regions, which are polygon entities. Both layers were imported into our software and displayed to create a base map of West Africa that clearly distinguishes the two administrative divisions (Figure 1). The Coordinate Reference System (CRS) was used.

#### 2.4.2. Spatio-Temporal Analysis

The layer resulting from our join was exported from QGIS software with the “.shp” extension to GeoDA version 1.8. First, we performed a global spatial autocorrelation test by comparing the standardized rates for each of our study years (2024 and 2025) to highlight the existence of a spatial dependence of MR vaccination campaigns within the countries studied. Second, we performed an autocorrelation with the Local Indicator of Space Association (LISA).

#### 2.4.3. Spatial Regression

Subsequently, we performed a spatial regression to determine if there was a spatial association following the use of different RR vaccination strategies depending on geographic location.

## 3. Results

### 3.1. Campaign Implementation

**Figure 2:**
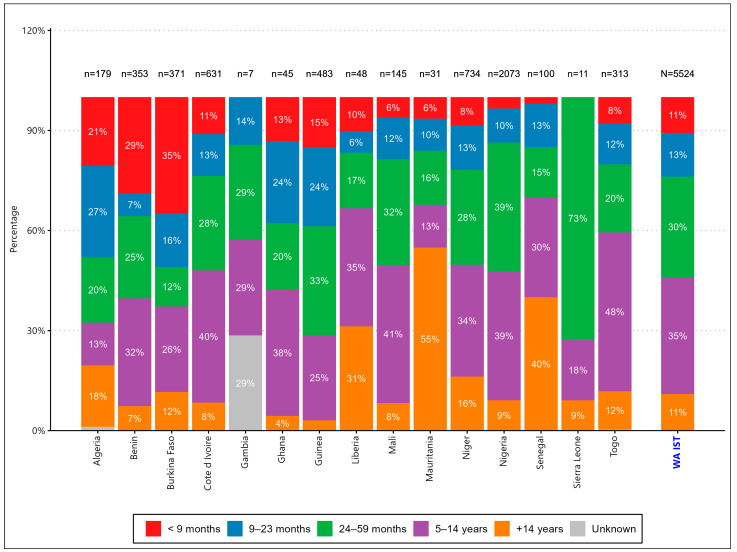
Confirmed cases of measles, by age group, in weeks 1 to 22 of the year 2025.

In 2024, a total of 12 out of 17 (70.5%) West Africa Inter-Country Support Team countries conducted national measles–rubella follow-up campaigns targeting children aged 9–59 months in 9 countries (Burkina Faso, Côte d’Ivoire, Ghana, Sierra Leone, Liberia, Guinea, Mauritania, Nigeria, and Benin), children aged 9 months–14 years in 2 countries (Mali and Guinea-Bissau), and children aged 9 months–15 years in 1 country (Senegal). Five countries (Algeria, Cape Verde, The Gambia, Niger, and Togo) did not conduct campaigns in 2024. A high concentration of confirmed measles cases was observed (Figure 1 and Figure 2) in the Sahel and border areas of Nigeria, Niger, Mali, and Burkina Faso between weeks 1 and 22 of 2025. These outbreaks coincided with areas of low vaccination coverage, revealing persistent vulnerability.

### 3.2. Monitoring of Country Readiness

The WHO preparedness assessment tool was used by countries to monitor pre-campaign preparedness levels to ensure quality campaigns. Pre-campaign preparedness levels in different countries are summarized in Table 1 (Preparedness Assessment for Immunization Campaigns in the 12 West African Countries, 2024–2025). Overall, all 12 countries (100%) used the WHO-recommended preparedness assessment tool at both the national and subnational levels (Region, Province, Department, District, Health Zone, and Commune, depending on the country). Of the 12 countries, half had above 80% preparedness at the start of their campaigns, and 6 others had below 80% preparedness (Burkina Faso, Guinea-Bissau, Mali, Mauritania, Nigeria, and Sierra Leone). This underperformance and the inability to complete or update dashboards in a timely manner remain a major challenge in most countries, delaying actions and increasing the risk of poor-quality campaigns.

### 3.3. Gaps in Vaccination Coverage

Cold-chain readiness was particularly weak in several countries. Guinea-Bissau (75%), Mauritania (25%), and Nigeria (50%) showed major gaps in refrigeration capacity, equipment maintenance, and last-mile transport. In Nigeria, the combination of large population size, extended distances, and inconsistent electricity supply contributed to poor cold-chain performance.

All countries, with the exception of Guinea-Bissau and Benin, reported administrative coverage below 95% (Table 2). No country reached 95% in the PCCS. The PCCS often revealed lower coverage than administrative data indicated, with the exception of Guinea-Bissau.

### 3.4. Trends in Epidemics

An epidemic occurs when a disease, health-related behavior, or other health-related event spreads unexpectedly or rapidly within a given geographic area or population.

**Figure 3:**
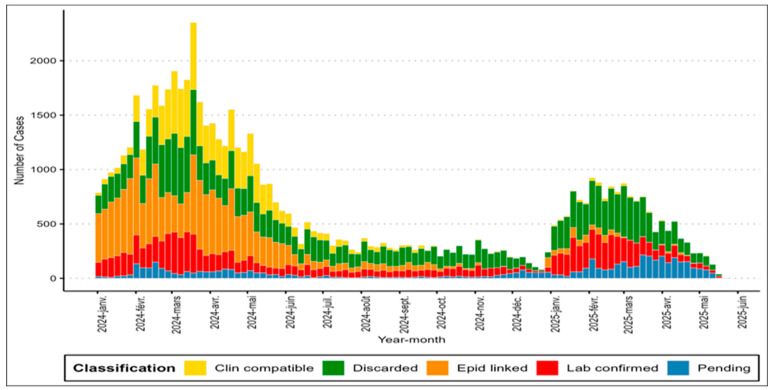
Colors represent weekly confirmed measles incidence levels across districts (light = low, dark = high).

Epidemiological trends (Figure 2) revealed that the number of confirmed cases decreased in 2025, but the number of epidemic districts increased from 64 to 383. In 2024, there were 64 epidemic districts (4% of the total) in 7 countries, while in 2025 (epidemiological week 16), there were 383 outbreaks (22%) in 13 countries (Figure 3).

## 4. Discussion

### 4.1. Main Conclusions in Context

Our results revealed three critical problems: a low overall level of preparedness of the country before the campaign, discrepancies between administrative and post-campaign coverage (coverage–quality paradox) and an age change in the epidemiology of measles. Although the follow-up MR campaigns showed important limitations, it is likely that measles outbreaks in 2025 would have been substantially worse without these campaigns. Several modeling studies suggest that supplementary immunization activities, even when imperfect, prevent large-scale accumulation of susceptible children and mitigate epidemic size.

**State of preparedness for measles and rubella vaccination campaigns in countries.**

As part of its assessment of measles vaccination campaign readiness, the WHO recommends that countries use the readiness assessment tool to implement a high-quality measles vaccination campaign. In the 12 African countries included in the study, readiness levels show that high reported levels of preparedness do not consistently lead to high-quality measles vaccination campaigns. This raises questions about the quality and accuracy of information provided by health actors at various levels.

This gap between the declared state of preparedness and the actual performance of campaigns constitutes a major challenge. This finding was also observed by Masresha et al. in their analysis of the performance of measles vaccination campaigns in the African Region, 2017–2021 [13]. This underscores that the reliability of the tool’s results depends on the data entered and the integrity of the processes it is intended to measure.

Monitoring and field supervision revealed several systemic problems contributing to this gap, including failure to meet the data completion deadline and the lack of updates to the readiness assessment tool data at both the national and subnational levels in some countries. Furthermore, delays in implementing preparatory activities according to the established timeline and the late availability of funds for the measles vaccination campaign, both nationally and internationally, are hindering the execution of key preparatory activities. These activities include meticulous micro-planning and timely advocacy and communication activities, which are essential for building community trust and ensuring strong participation. The identified delays correspond to the operational obstacles that can lead to the quality gaps observed in studies such as that by Masresha et al., ultimately threatening the goal of eliminating measles and rubella [13].

**The paradox of coverage and quality**

The resurgence of measles epidemics in 2025 West Africa Inter-Country Support Team countries, despite high administrative coverage reported in 2024, underscores the complexity of measles control. Our findings are consistent with global observations that vaccination coverage alone does not guarantee epidemic prevention [8]. Despite high administrative coverage (≥95% in 10/12 countries), the PCCS revealed substantial gaps (as low as 59.2% in Benin). This is consistent with studies showing that administrative data overestimate actual coverage by 15–20% in Africa [16]. These discrepancies are consistent with findings reported by Danovaro-Holliday et al. (2024) [14].

A recent study by Masresha et al. directly addresses this issue, analyzing the performance of measles vaccination campaigns in 28 African countries between 2017 and 2021. The authors found that while most countries reported high administrative coverage, the quality of the campaigns, as measured by the presence of good post-campaign coverage survey results, was inconsistent. Their research supports this concern, indicating that achieving the goal of measles elimination in the African region requires not only high coverage but also a high-quality vaccination campaign [13]. The phenomenon of “paper immunity,” where coverage exists on paper but not in communities, explains the persistent outbreaks in districts with high coverage [15,16,17].

Cold-chain failures likely contributed to reduced immunogenicity of vaccines in several countries. Inadequate refrigeration, limited ice-pack production, long transport distances, and frequent power interruptions in Guinea-Bissau, Mauritania, and Nigeria may have led to exposure of MR vials to temperatures outside the recommended range. Loss of potency due to heat exposure is a well-recognized risk factor for campaign failure in tropical settings.

The suboptimal quality of the campaigns, highlighted by discrepancies between administrative data and those from the Post-Campaign Coverage Survey (PCCS), further limits the impact. Similar concerns have been raised in other regions, where PCCS often reveals overestimation in administrative reports due to incomplete demographic data or human factors.

**Age change in the epidemiology of measles**

The predominance of cases among unvaccinated children and those of unknown status (85%) reflects persistent gaps in routine immunization and campaign reach. In particular, children aged 5–14 years accounted for 35% of cases, indicating that current age-based targeting may be insufficient to address immunity gaps. This supports calls to expand the age ranges of measles vaccination campaigns to include older children, especially in high-risk settings [2]. The 35% proportion of cases in the 5–14 age group mirrors trends observed in the DRC in 2023: 42% of cases in the 5–14 age group [18], and in India in 2022: outbreaks among school-aged children following campaigns focused on children under 5 [19]. This suggests that age-restrictive targeting creates immune gaps that fuel epidemics in older children.

**Spatial heterogeneity**

Outbreaks are clustered in border areas facing insecurity and cross-border migration: this is consistent with observations on mobile populations in the Sahel [20,21,22]. Furthermore, outbreaks occur in urban slums where population density exceeds the capacity of the vaccination system [23]. In addition, delayed campaigns during active outbreaks, such as those observed in Burkina Faso, Mali, and Algeria, can reduce effectiveness and contribute to continued transmission.

### 4.2. Political Implications

The 17 West African countries represent an estimated 438 million inhabitants in 2024, including approximately 53% under 18 years (≈232 million) and 16% under 5 years (≈70 million), based on UN population estimates.

These analyses suggest potential improvements in the design of MR campaigns in West African countries.

- Historically, most supplementary immunization activities in West Africa have targeted children aged 9–59 months because this age group accounts for the majority of measles mortality and because the cost–effectiveness profile is highest in early childhood. This policy originated from WHO recommendations in the early 2000s, when routine immunization was insufficient to protect infants. However, recurrent outbreaks among 5–14-year-olds in several countries raise the question of whether this age restriction should now be reconsidered. Expanding age targets: Data from Rwanda show that extending the target to 15 years reduced the number of cases by 64% [24]. Cost-effectiveness analyses suggest a reduction in marginal costs by leveraging school systems.
- Improve monitoring of preparedness for multiple measles outbreaks vaccination by leveraging experiences from polio vaccination campaigns in countries where high-quality immunizations have successfully halted transmission. This experience should be extrapolated to other vaccine-preventable diseases within the context of polio legacy.
- microplanning: Routine immunization campaigns appear to be vaccinating the same children who have already been vaccinated, raising questions about their effectiveness. Using historical data from health services and routine immunization programs can identify districts or provinces with high vaccination rates where microplanning can be implemented. Geospatial mapping can also be used to identify chronically under-immunized communities.

Data systems

The data system can be improved through real-time digital monitoring, as in Malawi, where the integration of DHIS2 reduced reporting errors by 70% [25,26,27]. Similarly, third-party PCCS, as in Ghana, improved data accuracy by 18% [23,28].

### 4.3. Limitations

- Surveillance variability: case detection differs between countries (e.g., Algeria’s robust system versus Niger’s weak reporting system)
- Gaps in genomic data: limited sequencing to confirm epidemic lineages
- Confounding factors: The prevalence of malnutrition (affecting seroconversion) has not been measured uniformly.

## 5. Conclusions

The 2024 measles vaccination campaigns failed to halt measles transmission in West Africa due to gaps in immunity, data inaccuracies, and suboptimal targeting. To improve the outcomes of measles vaccination campaigns, lessons learned from polio eradication efforts, such as digital surveillance tools, real-time monitoring, and community engagement, should be adapted. Strengthening surveillance systems and deploying technical assistance are essential to ensure campaigns reach unvaccinated children and achieve genuine herd immunity. Elimination requires broader age-targeting (up to 15 years).

### 5.1. What We Know About This Subject

- Measles remains one of the leading causes of preventable infant mortality in sub-Saharan Africa, despite the availability of a safe and effective vaccine.
- The two-dose regimen of the measles and rubella (MR) vaccine is highly effective (≥97% when administered on time) while approximately 85% of vaccinated children are protected after a single dose.
- The World Health Organization recommends periodic supplementary immunization activities (SIAs) every 3 to 4 years in countries to complement low routine immunization (<80%) and fill population immunity gaps and prevent endemic transmission of the measles virus.

### 5.2. What This Study Adds

- Post-campaign measles in West Africa has primarily affected children under five, the target of the campaigns, indicating poor quality of supplementary immunization activities against measles and rubella. Emphasis should be put on supporting countries during preparedness, improving data quality and field monitoring during campaign implementation
- The study reveals significant immunity gaps beyond early childhood, with 35% of cases occurring in children aged 5 to 14 years. This suggests that restrictive and age-limited targeting Measles Rubella mass campaigns in West African countries could inadvertently fuel outbreaks among the elderly and delay progress toward measles elimination.
- Despite the implementation of quality polio vaccination campaigns, countries have shown gaps in the implementation of measles immunization campaigns, highlighting the need to strengthen partner support and adapt lessons learned from polio to improve the preparedness and effectiveness of measles vaccination campaigns.
- Insecurity poses a fundamental threat to both measles elimination and polio eradication in West Africa by disrupting immunization services, weakening health systems, and displacing populations. Addressing immunization gaps in humanitarian contexts and integrating measles and Polio interventions is not just needed, it is essential.

## Figures and Tables

**Table 1:** Assessment of country preparedness prior to the implementation of Measles and Rubella vaccination campaigns in 12 West African countries, in 2024–2025.

Country	Planning and Coordination (%)	Monitoring and Supervision (%)	Vaccines, Cold Chain and Logistics (%)	Communication (%)	Funding (%)	Training (%)	Security (%)	Total Level (%)
Benin	100	100	90	96	33	58	99	95
Burkina Faso	100	33	91	0	0	0	80	72
Ivory Coast	100	100	98	75	64	88	100	95
Ghana	100	100	100	100	100	100	100	100
Guinea-Bissau	60	0	75	33	0	25	100	49
Guinea	100	100	100	75	63	88	100	95
Liberia	100	100	100	100	100	100	100	100
Mali	94	33	89	0	91	0	80	75
Mauritania	59	50	25	20	0	0	95	33
Nigeria	64	60	50	39	13	60	100	55
Senegal	100	91	89	70	58	85	100	94
Sierra Leone	84	44	65	9	0	0	100	65

**Table 2:** Comparison of administrative and post-campaign coverage of surveys in countries that have implemented the supplementary immunization activities MRI in West Africa.

Readiness Group	Country	Target Age Group	Target Population	Target Vaccinated	Administrative (%)	PCCS (%)	Difference (%)
<80% readiness	Burkina Faso	9–59 months	3,489,383	3,550,124	102	87	−15
	Guinea-Bissau	9 months–14 years	881,808	805,768	91	94	+3
	Mali	9 months–14 years	9,514,126	10,431,015	109	90	−19
	Mauritania	9–59 months	821,630	816,759	99	75	−24
	Nigeria	9–59 months	26,426,022	7,286,167	98	83	−15
	Sierra Leone	9–59 months	1,349,038	1,339,671	99	92	−7
≥80% readiness	Benin	9–59 months	3,732,669	3,115,648	83	60	−23
	Ivory Coast	9–59 months	6,846,061	6,723,826	98	71	−27
	Ghana	9–59 months	5,277,282	5,125,387	97	84	−13
	Guinea	9–59 months	3,705,438	3,728,686	101	N/A	N/A
	Liberia	9–59 months	779,789	781,124	100	81	−19
	Senegal	9 months–15 years	7,326,586	7,286,167	99	93	−6

## Data Availability

The data is available from the corresponding authors upon request from the editor-in-chief.
